# Content validity, interpretability, and internal consistency of the “Quality First” assessment to evaluate movement quality in hop tests following ACL rehabilitation. A cross-sectional study

**DOI:** 10.3389/fspor.2023.1180957

**Published:** 2023-06-16

**Authors:** Moritz Mathieu-Kälin, Mirjam Müller, Melanie Weber, Sandro Caminada, Marina Häberli, Heiner Baur

**Affiliations:** ^1^Department of Physiotherapy, School of Health Professions, Bern University of Applied Sciences, Bern, Switzerland; ^2^Altius Swiss Sportmed Center, Rheinfelden, Switzerland

**Keywords:** anterior cruciate ligament (ACL), movement quality, return to sport (RTS), measurement properties, hop test, assessment

## Abstract

**Introduction:**

Current approaches fail to adequately identify sport readiness after anterior cruciate ligament (ACL) rehabilitation. Altered landing biomechanics after ACL reconstruction are associated with increased risk of a noncontact ACL reinjury. There is a lack of objective factors to screen for deficient movement patterns. Therefore, the aim of this study was to investigate content validity, interpretability, and internal consistency for the newly developed “Quality First” assessment to evaluate movement quality during hop tests in patients after ACL rehabilitation.

**Method:**

Participants in this cross-sectional study were recruited in collaboration with the Altius Swiss Sportmed Center in Rheinfelden, Switzerland. After a successful ACL reconstruction, the movement quality of 50 hop test batteries was evaluated between 6 and 24 months postoperatively with the “Quality First” assessment. Content validity was assessed from the perspective of professionals. To check the interpretability, classical test theory was employed. Cronbach's *α* was calculated to evaluate internal consistency.

**Results:**

Content validity resulted in the inclusion of three different hop tests (single-leg hop for distance, vertical hop, and side hop). The “Quality First” assessment is enabled to evaluate movement quality in the sagittal, vertical, and the transversal plane. After the exclusion process, the “Quality First” assessment was free from floor and ceiling effects and obtained a sufficient Cronbach's *α*. The final version consists of 15 items, rated on a 4-point scale.

**Discussion:**

By means of further validations, the “Quality First” assessment could offer a possibility to evaluate movement quality after ACL rehabilitation during hop tests.

## 1. Introduction

Return to sport (RTS) outcomes following anterior cruciate ligament (ACL) reconstruction are unsatisfactory (1), whereas current approaches fail to adequately identify sport readiness (2). An underestimated factor may be altered landing biomechanics after ACL reconstruction (3–5), which are associated with increased risk of a noncontact ACL reinjury (3, 6). Achieving symmetrical results in the single-leg hop for distance (SLHD) test does not ensure symmetry in kinematic variables (7, 8). Therefore, the evaluation of movement quality should be included besides quantitative and psychological parameters in RTS assessments after ACL reconstruction (1, 9–11).

Three-dimensional motion analysis as the gold standard to capture biomechanical risk factors for a reinjury of the ACL is not feasible for most clinical settings because of financial, spatial, and temporal costs (4, 12). There is a lack of objective factors to screen for deficient movement patterns (4). The Landing Error Scoring System is a simple tool to identify potentially high-risk movement patterns during a bipedal jump-landing task (13–15).

Bipedal assessments may not be sensitive enough to identify asymmetries in lower extremity (16). In addition, single-leg landings are a typical ACL injury mechanism (17). Consequently, single limb tasks such as single-leg hop tests are important to identify limb asymmetries in movement and landing patterns (18).

A recent systematic review did not reveal an effective assessment to evaluate movement quality during hop tests after ACL reconstruction (15). With this background and an in-depth literature search about the biomechanical risk factors for ACL injuries, our work group created the assessment tool “Quality First”. With this assessment under examination, the work group expects to reliably identify deficient landing patterns during single-leg hop tests. To account for the variable injury mechanisms the “Quality First” assessment was developed to evaluate movement quality during landings of the SLHD, the vertical hop (VH), and the side hop (SH) test (19).

To ensure that meaningful data can be extracted from the presented assessment during hop tests after ACL reconstruction, the measurement properties of the tool must be established (20). The aim of this study was to investigate content validity, interpretability, and internal consistency for the “Quality First” assessment during hop tests in patients after ACL reconstruction.

## 2. Methods

### 2.1. Study design

This study corresponded to a cross-sectional study design (21) and adhered to the STROBE guidelines (22). The movement quality during hop tests of the participants was evaluated at a specific time point with the “Quality First” assessment.

### 2.2. Participants

Participants in this study were recruited in collaboration with the Altius Swiss Sportmed Center in Rheinfelden, Switzerland. After a successful ACL reconstruction, patients of this clinic routinely attended a RTS test battery between 6 and 24 months postoperatively. Fifty RTS single-leg test batteries from 27 participants were included. Each test leg was evaluated individually. Only participants who were able to hop unilaterally with each leg were included. If the performance of one leg was incorrect, only the test from the correct leg was included. All inclusion and exclusion criteria are shown in Table 1.

**Table 1:** Inclusion and exclusion criteria.

Inclusion criteria	Exclusion criteria
• Time from ACL reconstruction 6–24 months • Attend routine RTS test battery in Altius Swiss Sportmed Center	• Single-leg hopping not possible • Incorrect test procedure (based on instruction document (Supplementary Figure S2) • Incomplete RTS test (at least one of the three hop tests missing)

ACL, anterior cruciate ligament; RTS, return to sport.

The patient characteristics are shown in Table 2. The age ranged from 14 to 39 years, 40.7% were female, and the average body mass index (BMI) was 23.7. The mean time since surgery was 10.1 months. In 85.2% and 14.8% of the participants, semitendinosus and quadriceps, respectively, were used as autografts. In three cases, the ACL reconstruction was a revision surgery. Some participants had associated injuries on the meniscus (*n* = 17), ligaments (*n* = 3), cartilage (*n* = 4), or others (femoral notchplasty, *n* = 2). As visible in the mean Tegner score (7.5), participants mostly participated in competitive sports before surgery.

**Table 2:** Patient characteristics.

Characteristics (*n* = 27)	Value
Age, y (m ± SD)	24.2 (±6.5)
Sex, *n* (%)	
Women	11 (40.7)
Men	16 (59.3)
Height, cm (m ± SD)	175.4 (±7.6)
Weight, kg (m ± SD)	73.3 (±12.0)
BMI, kg/m^2^ (m ± SD)	23.7 (±2.7)
Injured knee, *n* (%)	
Right	12 (44.4)
Left	15 (55.6)
Time since surgery, M (m ± SD)	10.1 (±3.1)
ACL revision surgery, *n* (%)	3 (11.1)
Type of graft, *n* (%)	
Semitendinosus	23 (85.2)
Quadriceps	4 (14.8)
Associated injuries, *n* (%)	
Meniscus	18 (66.7)
Ligament	3 (11.5)
Cartilage	4 (15.4)
Other	2 (7.7)
Tegner score before injury (m ± SD)	7.5 (±1.0)

ACL, anterior cruciate ligament; BMI, body mass index; M, month; m, mean; *n*, number; SD, standard deviation; y, year.

All participants gave their written general consent prior to participation. The study was evaluated by the Regional Ethical Review Board of “Nordwest- und Zentralschweiz” (Project-ID: 2021–01169).

### 2.3. Data collection

The “Quality First” construct seeks to describe the latent variable “movement quality” in the form of a reflective model and corresponds to a 66-point scale with three subgroups. A 4-point scale was used to represent different characteristics while maintaining a high discriminability within the items (23). In total, 22 items were rated between 0 and 3. Therefore, the total score ranged between 0 and 66 points. A higher number of points indicated a better movement quality. The subgroups consisted of eight items from the SLHD, the same eight items from the VH, and six items from the SH test. The maximal score for the individual subgroups was 24 for the SLHD and the VH test, and 18 for the SH test. The items included general characteristics like shock absorption during landing and joint-specific movement quality parameters of the trunk, hip, knee, and the foot. An example of the knee evaluation was the item “knee alignment”, where the movement quality was rated from 0 (“The knee joint is neutrally aligned with the axis during landing”) to 3 (“Extreme knee joint valgus”). Full description of the “Quality First” assessment is available in the Supplementary Figure S1).

Data collection took place between 2021 and 2022. After participation was confirmed, SLHD, VH, and SH tests were performed with each leg on the same occasion. For SLHD and VH, three test trials were performed. In case the last trial was the best, the test continued until no improvements were made. For conduction of the SLHD test, participants jumped as far as possible on one leg and landed with the same leg. During the VH test, participants jumped as high as possible on one leg and landed on the same leg. For SLHD and VH tests, the hands of the participants were unconstrained. To generate a valid test, the landing position needed to be held stable for at least 2 s. For the SH, participants jumped on one leg from side to side over two 40 cm apart parallel strips. With their hands placed on their hips, participants were required to jump during 30 s as many times as possible over the two strips, without touching the marking line. For the SH test, only one test trial was performed. The test had to be repeated if more than 25% of the jumps were failed attempts. For each of the three hop tests, the uninjured leg was tested first. Two independent sport scientists from the Altius Swiss Sportmed Center were instructed to videotape the tests in a standardized manner with an iPad® Pro 11.0 (Apple Inc., Cupertino, CA, United States) from a frontal view and to label them with the patient ID (Supplementary Figure S2). Through the Dartfish®-Application (Dartfish® 360, Dartfish HQ, Lausanne, CH), the anonymized video tapes were recorded with a frame rate of 120 frames per second and a resolution of 1080p (pixels) and then uploaded to a secured cloud system, where the examiners evaluated the movement quality. Three physical therapists independently observed the videos several times and in slow motion without any time limit. They could watch the video as often as necessary, until they finalized their score with the “Quality First” assessment to rate the movement quality. Later, consensus was made by the same physical therapists and used for statistical calculations in this study.

### 2.4. Data analysis

The COSMIN guidelines were used to evaluate measurement properties of the “Quality First” assessment (20). The present study was a first step in validating the “Quality First” assessment in participants after ACL reconstruction including content validity, interpretability (floor and ceiling effects), and internal consistency.

### 2.5. Statistical methods

#### 2.5.1. Content validity

Content validity was assessed from the perspective of professionals (20). Three physical therapists who were candidates for a master's degree in sports physiotherapy with 3–4 years of clinical experience in sports physiotherapy created the “Quality First” assessment with current biomechanical risk factors for ACL injuries based on an internal structured literature search in 2020. The relevance of the included hop tests and the individual items were discussed in this work group with a sports scientist who had a doctoral degree. The structured developing process included a first field test with seven physical therapists working in two different outpatient clinics. After a consensus discussion and an improvement procedure, a second field test was conducted by the work group. The resulting version was finalized in a second consensus meeting between the work group and the sports scientist.

#### 2.5.2. Interpretability

To check if all response options were informative, the classical test theory was employed. The distribution of the score at the item level determined to what extent the response options were used and were presented with frequency tables (24). Floor and ceiling effects occurred, if more than 15% of the participants achieved the lowest or highest possible score of a subgroup (24).

#### 2.5.3. Internal consistency

Internal consistency was assessed to show the degree of the interrelatedness among the individual items and their subgroup (20). Cronbach's *α* (25) was calculated for each subgroup separately to estimate item-specific variance (26). To explore if a subgroup should be excluded from the assessment, a Cronbach's *α* between 0.7 and 0.95 was considered adequate (27). Furthermore, Spearman’s rank correlation between the items and the subgroup was calculated. Items from a subgroup not attaining a Cronbach's *α* between 0.7 and 0.95 were excluded if (1) the Cronbach's *α* value increased when the item was excluded and (2) the item–subtotal correlation was below 0.2 or higher than 0.7 (24). The work group decided about the exclusion of a subgroup with Cronbach's *α* values between 0.65 and 0.7 and values higher than 0.95 (28). Furthermore, the difficulty and discrimination of the individual items were presented. All statistical analyses were carried out using the program RStudio (Version 4.1.2, License AGPL v3, Boston, MA, United States). Only complete RTS tests were included; therefore, missing data did not have to be addressed.

## 3. Results

### 3.1. Participants

In 4 out of the 27 included participants, only one leg (2 affected, 2 unaffected) was evaluated. This was based on incorrect procedure or incomplete test battery of the second leg. The decision to include unaffected legs was based on the opportunity to ensure more heterogeneity within the jump performances.

### 3.2. Content validity

The work group discussion resulted in the inclusion of three different hop tests [SLHD, VH, and SH (19)]. With those three subgroups, the “Quality First” assessment is enabled to evaluate movement quality in the sagittal, vertical, and transversal planes. Furthermore, the 30 s time duration of the SH test adds the component of fatigue and possible deterioration of movement quality over time. The work group excluded the item “trunk flexion” because of the difficulty to discriminate between trunk and hip flexion in a 2D frontal view. After the first field test, the items “hip tilt”, “hip flexion”, and “knee flexion” were excluded in the SH test subgroup due to different biomechanics during a frontal plane hop test. The results of the second field test were used to improve the written explanations of the different items (Supplementary Figure S1).

### 3.3. Interpretability

Distribution of the score at the item level is presented with frequency tables (Table 3). The mean scores ranged between 1.7 and 2.8. In terms of the subtotal scores, neither floor nor ceiling effects were observed (Supplementary material Figures S3–S5).

**Table 3:** Distribution of the score at item level.

Subgroup individual items (%)	0	1	2	3	Mean	±SD
**VH**						
Shock absorption	0	10	44	46	2.4	0.7
Trunk lateral flexion	0	8	72	20	2.1	0.5
Hip rotation	0	26	52	22	2.0	0.7
Hip tilt	2	24	58	16	1.9	0.7
Hip flexion	2	14	34	50	2.3	0.8
Knee alignment	4	24	48	24	1.9	0.8
Knee flexion	0	2	34	64	2.6	0.5
Foot position	0	14	56	30	2.2	0.7
**SLHD**						
Shock absorption	0	26	58	16	1.9	0.7
Trunk lateral flexion	0	22	66	12	1.9	0.6
Hip rotation	4	18	52	26	2.0	0.8
Hip tilt	2	20	74	04	1.8	0.5
Hip flexion	0	14	34	52	2.4	0.7
Knee alignment	4	22	58	16	1.9	0.7
Knee flexion	0	0	20	80	2.8	0.4
Foot position	0	2	70	28	2.3	0.5
**SH**						
Shock absorption	0	4	40	56	2.5	0.6
Trunk lateral flexion	0	16	44	40	2.2	0.7
Hip rotation	0	26	70	4	1.8	0.5
Knee alignment	2	36	50	12	1.7	0.7
Foot position	2	40	44	14	1.7	0.7
Fatigue	4	28	44	24	1.9	0.8

SD, standard deviation; SH, side hop; SLHD, single-leg hop for distance; VH, vertical hop.

### 3.4. Internal consistency

The total Cronbach's *α* coefficient (0.72) was considered adequate, and therefore, no complete subgroup was excluded (Table 4).

**Table 4:** Internal consistency of the “Quality First” assessment.

Subgroup	Mean	±SD	Subgroup difficulty	Subgroup discrimination	Cronbach's *α*	*α* if deleted
VH	17.3	2.9	0.75	0.56	0.72	0.61
SLHD	16.9	2.4	0.80	0.47		0.70
SH	11.8	2.4	0.74	0.59		0.56

SD, standard deviation; VH, vertical hop; SLHD, single-leg hop for distance; SH, side hop.

Cronbach's *α* of the subgroup analysis ranged from 0.58 to 0.65, the item difficulty from 0.07 to 0.87, and the item discrimination from 0.04 to 0.66 (Table 5). Based on the improvement of the subgroups’ Cronbach's *α*, if an item was deleted, the following items were excluded in a first step: “trunk lateral flexion” in the VH and SLHD tests, “hip flexion” and “foot position” in the SLHD test, and “tiredness” in the SH test. In the second step, the items “shock absorption” and “knee flexion” of the SLHD were additionally excluded (Supplementary Figure S6).

**Table 5:** Subgroup analysis (items to be deleted in italic).

Subgroup	Item difficulty	Item discrimination	Cronbach's *α*	*α* if deleted
VH			0.65	
Shock absorption	0.79	0.26		0.63
*Trunk lateral flexion*	0.71	0.09		0.66
Hip rotation	0.65	0.66		0.52
Hip tilt	0.63	0.42		0.59
Hip flexion	0.77	0.31		0.62
Knee alignment	0.64	0.42		0.59
Knee flexion	0.87	0.29		0.63
Foot position	0.72	0.25		0.63
SLHD			0.58	
*Shock absorption*	0.37	0.29		0.54
*Trunk lateral flexion*	0.37	0.04		0.61
Hip rotation	0.67	0.46		0.47
Hip tilt	0.60	0.40		0.51
*Hip flexion*	0.21	0.11		0.60
Knee alignment	0.62	0.55		0.44
*Knee flexion*	0.07	0.36		0.53
*Foot position*	0.75	0.11		0.59
SH			0.63	
Shock absorption	0.84	0.39		0.59
Trunk lateral flexion	0.75	0.37		0.59
Hip rotation	0.59	0.50		0.56
Knee alignment	0.57	0.50		0.54
Foot position	0.57	0.27		0.63
*Fatigue*	0.63	0.26		0.64

VH, vertical hop; SLHD, single-leg hop for distance; SH, side hop.

Spearman’s rank correlation at the item–subgroup level ranged from 0.19 to 0.77 (Supplementary Figures S7–S9). “Trunk lateral flexion” (item–subgroup correlation: 0.19) was excluded from the SLHD test, and “hip rotation” (item–subgroup correlation: 0.77) was retained due to a sufficient Cronbach's *α* of the VH test. All other items met a requested item–subtotal correlation (Supplementary Figures S7–S9).

### 3.5. Final version of the “Quality First” assessment

The final version of the “Quality First” assessment consists of the VH (“shock absorption,” “hip rotation,” “hip tilt,” “hip flexion,” “knee alignment,” “knee flexion,” and “foot position”), the SLHD (“hip rotation,” “hip tilt,” and “knee alignment”), and the SH (“shock absorption,” “trunk lateral flexion,” “hip rotation,” “knee alignment,” and “foot position”). Cronbach's *α* from 0.64 to 0.66 was considered sufficient after a work group consensus discussion (VH, SLHD, and SH; Table 6). The possible subgroup scores of the final 45-point scale version are 21 for the VH, 9 for the SLHD, and 15 for the SH test (Supplementary Figure S10).

**Table 6:** Final version of the “Quality First” assessment.

Subgroup	Item discrimination	Cronbach's *α*	*α* if deleted
VH		0.66	
Shock absorption	0.27		0.66
Hip rotation	0.64		0.54
Hip tilt	0.38		0.62
Hip flexion	0.37		0.63
Knee alignment	0.41		0.62
Knee flexion	0.30		0.65
Foot position	0.25		0.66
SLHD		0.78	
Hip rotation	0.66		0.65
Hip tilt	0.42		0.88
Knee alignment	0.83		0.43
SH		0.64	
Shock absorption	0.29		0.63
Trunk lateral flexion	0.39		0.60
Hip rotation	0.52		0.55
Knee alignment	0.52		0.53
Foot position	0.32		0.64

VH, vertical hop; SLHD, single-leg hop for distance; SH, side hop.

## 4. Discussion

This study aimed to investigate the measurement properties of the “Quality First” assessment during hop tests in patients after ACL reconstruction. The final version was free from floor and ceiling effects, obtained a sufficient Cronbach's *α*, and showed adequate item–subgroup correlations.

In the process to determine content validity, every item was selected due to its relevance as a risk factor for an ACL reinjury. This process was led through a structured literature research, clinical expertise, and discussions in the work group with three physical therapists and a movement scientist. Content validity has been investigated in a similar way (28, 29). In one study, four clinicians were asked to rank five functional tasks about their usefulness regarding the degree of knee flexion (29). In another study, a work group of three clinicians and a focus group including two more experts discussed and determined tasks and postural orientation errors based on current scientific knowledge and clinical experience (28). Based on the reported results, the presented procedure seems to be reasonable to establish sufficient content validity for the “Quality First” assessment.

In terms of the interpretability, neither floor nor ceiling effects were observed in the subgroup scores. Regarding the floor effects, it must be considered that only patients with the ability to perform all three hop tests in a safe execution were included in this study. If a patient is not able to perform hop tests in all movement directions, return to sport clearance should not be considered. A comparable study with a similar scale detected several floor effects using a different definition (>70%) on the item level (28). In the present study, floor and ceiling effects were calculated on the subgroup level, because movement quality consists of the interaction of each item. Each item is meant to discriminate between the performance of one aspect in movement quality and is allowed to contain floor or ceiling effects due to different item difficulties (24).

Regarding the internal consistency, the subgroups of the final version reached a sufficient Cronbach's *α* (VH: 0.66, SLHD: 0.78, SH: 0.64). A similar study calculated a comparable Cronbach's *α* (0.82) for postural orientation errors during the SLHD (28). In a subsequent study, an SH task attained a Cronbach's *α* of 0.64 for the lateral and 0.82 for the medial landing (30). This SH task was performed in a self-selected pace and with only seven landings instead of the 30 s maximal procedure in this study. The work group of the present study considered the calculated Cronbach's *α* as adequate due to its dependency upon the number of items in a scale, the homogeneity of the participants, and the fundamental differences between the individual items (26, 31).

The exclusion of the item “trunk lateral flexion” in the VH and SLHD tests can be justified through its unclear role as a risk factor for a second ACL injury. A meta-analysis stated moderate evidence that trunk lateral flexion in SLHD landings after an ACL reconstruction does not differ compared with a healthy control group (7). This item sustained in the final version of the SH may be due to the changes of direction during the test procedure. In this context, an ACL reinjury group showed greater asymmetry of trunk lateral flexion during a change of direction task, compared with a no-reinjury group (11). In comparison with the contralateral leg, another study indicated strong evidence for no difference in peak hip flexion, which could justify the exclusion of the item “hip flexion” in the SLHD test (7). Like the other excluded items, “foot position” in the SLHD showed low item discrimination (0.11) and therefore fails to influence the subgroup score (23). The work group accounted for the exclusion of the item “fatigue” during the SH due to the difficulty in rating this item. Despite this exclusion, the strength of the SH test to evaluate possible deteriorations of movement quality over time sustains in the other five items of this subgroup. The decision to exclude the items “shock absorption” and “knee flexion” from the SLHD in a second step was underpinned due to their negative correlation with the total scale. Two items did not meet the desired item–subgroup Spearman rank correlation between 0.2 and 0.7. The item “trunk lateral flexion” would have been excluded anyways due to an improvement in the subgroup Cronbach's *α* of the SLHD test, and the item “hip rotation” remained in the final version based on a work group consensus discussion. The conclusion of this discussion was based on the fact that excessive internal hip rotation angles during landings are thought to increase the ACL injury risk (32).

The “Quality First” assessment is the first measurement tool to evaluate movement quality during a hop test battery consisting of SLHD, VH, and SH tests. This tool would allow a clinically friendly ready-to-use approach to include the movement quality in an RTS test after ACL reconstruction. The hop test performance for quantitative measures is simply videotaped with an iPad or a similar device and can be analyzed with a common slow-motion viewer. Before the “Quality First” assessment should be used in clinical practice, further investigations are needed. There is one study in progress that examines the inter- and intrarater reliability and to analyze the correlations between the classic quantitative measures and the movement quality outcomes. Another study under way explores the inter- and intrarater reliability of a real-time execution in contrast to the slow-motion analysis of the “Quality First” assessment.

A current limitation of this study is that the structural validity, construct validity, and responsiveness were not assessed as recommended (20). Those limitations should be addressed in future studies. Another future project is needed to focus on the ability of the “Quality First” assessment to evaluate the risk of a possible reinjury in a long-term follow-up study.

It must be pointed out that the implementation of such a quality control instrument in clinical practice warrants specific training and sound instructions for the users in practice. This is especially important when already test batteries have been applied and those routines have to be changed by adding new and more standardized elements. Due to these issues and the inability to perform one of the hop tasks, an unknown number of participants from the routine sample of patients were excluded.

The “Quality First” assessment could offer a possibility to evaluate movement quality after ACL rehabilitation to guide RTS decisions together with a combination of strength, and other performance measures, psychological readiness, and nonphysical factors. By means of further validations, this assessment could be used to provide the patient with important feedback regarding their readiness to RTS. Additionally, the “Quality First” assessment could lead to tailor specific interventions based on detected deficiencies of the individual patient after ACL rehabilitation.

## Data Availability

The original contributions presented in the study are included in the article/Supplementary Material, further inquiries can be directed to the corresponding author.
