# Supplementary material for: Content validity, interpretability, and internal consistency of the “Quality First” assessment to evaluate movement quality in hop tests following ACL rehabilitation. A cross-sectional study
Source: Front Sports Act Living. 2023 Jun 16;5:1180957. doi: 10.3389/fspor.2023.1180957 (PMC10313111; doi:10.3389/fspor.2023.1180957)
Supplement: Supplementary file 1 [file Datasheet1.docx]

Supplementary Material

Content Validity, Interpretability, and Internal Consistency of the «Quality First» Assessment to Evaluate Movement Quality in Hop Tests Following ACL Rehabilitation. A Cross-Sectional Study

Moritz Mathieu-Kälin^1*^, Mirjam Müller^1^, Melanie Weber^1^, Heiner Baur^1^

*** Correspondence:** Moritz Mathieu-Kälin, moritz.kaelin@outlook.com

# Supplementary Data

Supplementary Figure 1. “Quality First” assessment

Supplementary Figure 2. Procedure of the Hop Tests

Supplementary Figure 3. Frequency table of the VH (vertical hop)

Supplementary Figure 4. Frequency table of the SLHD (single leg hop for distance)

Supplementary Figure 5. Frequency table of the SH (side hop)

| Subgroup | Item discrimination | Cronbach’s α | α if deleted |
| --- | --- | --- | --- |
| SLHD  *Shock absorption*  Hip rotation  Hip tilt  Knee alignment  *Knee flexion* | 0.20  0.56  0.44  0.66  0.27 | 0.66 | *0.71*  0.53  0.60  0.47  *0.67* |

Supplementary Figure 6. Second exclusion process of the SLHD (single leg hop for distance) (items to be deleted in italic)


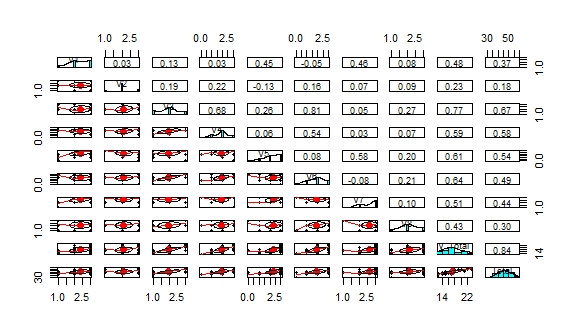


Supplementary Figure 7. Pairs panels of the VH (vertical hop)


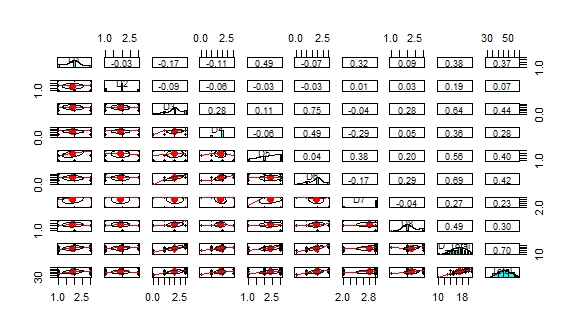


Supplementary Figure 8. Pairs panels of the SLHD (single leg hop for distance)


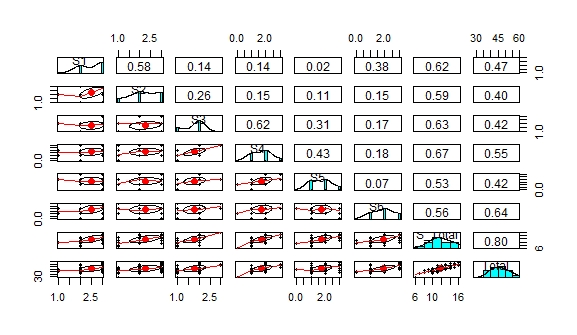


Supplementary Figure 9. Pairs panels of the SH (side hop)

Supplementary Figure 10. Final version of the “Quality First” assessment

Supplementary Figure 11: Dataset
